# *Helicobacter pylori* infection reduces TAMs infiltration in a mouse model of AOM/DSS induced colitis-associated cancer

**DOI:** 10.1371/journal.pone.0241840

**Published:** 2020-11-17

**Authors:** Luo-na Li, Yun Liu, Hong-chen Zhang, Ting Wu, Yun Dai, Wei-hong Wang

**Affiliations:** Department of Gastroenterology, Peking University First Hospital, Beijing, China; Icahn School of Medicine at Mount Sinai, UNITED STATES

## Abstract

Inflammatory bowel disease (IBD) increases the risk of colitis-associated cancer (CAC). Evidences suggest that *Helicobacter pylori* (*H*. *pylori*) infection is associated with a low risk of IBD and protects against experimental colitis in mouse models. However, the effect of *H*. *pylori* infection in CAC remains unclear. We previously reported that *H*. *pylori* infection increased M2 macrophages in dextran sodium sulfate (DSS)-induced chronic colitis. Tumor-associated macrophages (TAMs) play a pivotal role in colon cancer. Therefore, we established a *H*. *pylori*-infected CAC mouse model induced by azoxymethane and DSS to explore the effect of *H*. *pylori* infection on TAMs in CAC. Here, we demonstrated that *H*. *pylori* infection attenuated the development of CAC by decreasing tumor multiplicity, tumor size, tumor grade and colitis scores. Moreover, *H*. *pylori* infection reduced the infiltration of TAMs, particularly M2-like TAMs in CAC tumors, accompanied with the down-regulated pro-inflammatory and pro-tumorigenic factors TNF-α, IL-1β, IL-6 and IL-23 in tumors of CAC mice. Our study suggests that *H*. *pylori* infection can reduce TAMs infiltration and regulate cytokines expression in CAC.

## Introduction

Colitis-associated cancer (CAC), associated with inflammatory bowel disease (IBD), only accounts for 1–2% of all colorectal cancer (CRC) cases in general population [1]. The risk of CAC increases with the duration and anatomic extent of inflammation [2–4]. Large epidemiological studies have demonstrated a low risk for IBD in patients with *Helicobacter pylori* (*H*. *pylori*) infection. Furthermore, animal studies have revealed the protective effect of *H*. *pylori* infection in acute and chronic colitis [5, 6]. However, some epidemiological studies and the latest meta-analysis have shown that CRC is associated with *H*. *pylori* infection [7, 8]. Therefore, the effect of *H*. *pylori* infection in CAC remains unclear.

Unlike the typical “adenoma-adenocarcinoma” process of sporadic CRC, CAC follows the sequence of “inflammation-dysplasia-adenocarcinoma” [4]. Moreover, in CAC tumorigenesis, the immunological mechanisms are more involved and immune cells interact with tumor cells [9]. We previously reported that *H*. *pylori* infection alleviated dextran sodium sulfate (DSS)-induced chronic colitis through balancing T helper cells 17 / regulatory T cells responses and increasing M2 macrophages [10]. However, the effect of *H*. *pylori* infection on macrophages in CAC is still unknown.

Macrophages have a critical role in inflammation and tumor [11, 12]. Usually, activated macrophages are divided into classical M1 and alternative M2 macrophages, based on their function. M1 macrophages express high levels of pro-inflammatory cytokines (e.g. TNF-α, IL-1β, IL-6 and IL-23) and are considered to exert tumoricidal effects. In contrast, M2 macrophages are involved in anti-inflammatory responses and tumor-promoting activities [13]. Tumor-associated macrophages (TAMs) refer to macrophages within tumor and closely resemble M2 macrophages, which are related to promote tumor growth and metastasis [14].

Considering that M2 macrophages have the different influences on inflammation and tumor, in this study, we established a *H*. *pylori*-infected CAC mouse model induced by azoxymethane (AOM)/DSS, aiming to explore the role of *H*. *pylori* infection on TAMs, especially on M2-like TAMs in CAC.

## Materials and methods

### *H*. *pylori* colonization and CAC induction

**Fig 1:**
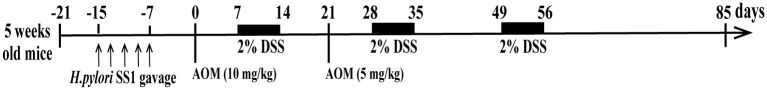
Schematic of the animal treatment.

Male 5 weeks old C57BL/6 mice were purchased from Beijing Vital River Laboratory Animal Technology Company Limited, China. The mice were adapted to the environment for 1 week before entering the formal study. All mice were randomly divided into 4 groups: the control group (control), *H*. *pylori*-infected group (Hp), AOM/DSS-treated group (AOM/DSS), and AOM/DSS-treated with *H*. *pylori* infection group (Hp+AOM/DSS). In the Hp and Hp+AOM/DSS groups, 6-week-old mice fasted overnight were intragastrically inoculated 5 times with 200 μl brain-heart infusion containing 1×10^9^ colony-forming units per ml *H*. *pylori* SS1 every other day. Mice in control and AOM/DSS groups were inoculated with brain-heart infusion without *H*. *pylori* SS1. One week later, CAC was induced as the protocol previously described [15]. Mice in AOM/DSS and Hp+AOM/DSS groups were intraperitoneally injected with AOM (10 mg/kg, Sigma-Aldrich, USA) on day 0. One week after AOM injection, mice were given 2% DSS (MP Biomedicals, USA) in drinking water for 7 days followed by substitution of regular water for 14 days. During this time, mice received a second intraperitoneal injection of AOM (5 mg/kg) on day 21. Then, mice were subjected to 2 more cycles of 2% DSS treatment (7 days/cycle). Mice were sacrificed on day 85 after AOM/DSS challenge (Fig 1). All animal experiments were reviewed and approved by the Animal Studies Committee of Peking University First Hospital (Protocol Number: J201823).

### Histopathological and immunohistochemical analyses

Histopathological appearances in colonic tissues were determined by hematoxylin and eosin (H&E) staining. The criteria of colitis score in non-tumor areas was described previously [16] as follows: severity scores for inflammation (0, normal; 1–3, mild-serve), scores for ulceration (0, normal; 1–3, mild-serve), scores for mucosal hyperplasia (0, normal; 1–3, mild-serve), and scores for extent of lesions (0, normal; 1–3, mild-serve). The score of each part was added to give a total histological score. For immunohistochemistry, the polyclonal rabbit anti-*H*. *pylori* antibody (1:250, Abcam, UK) was staining in gastric tissues to identify the colonization of *H*. *pylori* and the expression of F4/80, CD206 and CD31 in colon tissues was detected respectively using monoclonal rabbit anti-F4/80 antibody (1:300, Cell Signaling Technology, USA), polyclonal rabbit anti-CD206 (1:10000, Abcam, UK) and monoclonal rabbit anti-CD31 antibody (1:100, Cell Signaling Technology, USA). Each slide was randomly selected 5 high power fields in tumor sections (×400) and was calculated the average positive staining cells per filed.

### Cell isolation and flow cytometry

Colonic Lamina propria mononuclear cells were isolated from fresh colonic tissues as described previously [17]. Colonic tissues were cut into small pieces and incubated twice in Hank’s Balanced Salt Solution (HBSS) containing 5 mM EDTA and 1 mM DTT for 20 min at 37 °C under slow rotation. After washing off the EDTA, the remaining tissues were cut into 1mm pieces and incubated twice in digestion solution containing 0.5 mg/ml DNase I and 0.5 mg/ml collagenase IV and 3 mg/ml dispase II (all from Sigma Aldrich, USA) for 20 min at 37 °C. The cell solution was filtered through a 70-μm cell strainer and then layered on a percoll density gradient to separate immunocytes. For macrophages surface staining, the mononuclear cell suspensions were incubated with antibodies on ice for 30 minutes, and analyzed on a BD Influx^™^ (BD Biosciences, USA). The following monoclonal rat anti-mouse antibodies were used: anti-CD45-APC/Cy7, anti-CD11b-PE/Cy7, anti-F4/80-PE, anti-CD206-APC (all from BioLegend, USA). In parallel, isotype antibodies were used as control. The results were analyzed using Flow Jo 7.6 software (Tree Star, USA).

### Magnetic-activated cell sorting (MACS)

The lamina propria mononuclear cell were obtained from colon tissues as described above. TAMs were purified from these mononuclear cells using Anti-F4/80 MicroBeads UltraPure (Miltenyi Biotec, Germany) followed by magnetic separation.

### Quantitative real-time PCR (qPCR)

Total RNA from colonic tissues or TAMs was extracted using Trizol reagent (Invitrogen, USA) and reverse-transcribed to cDNA using High-Capacity cDNA Reverse Transcription kits (Applied Biosystems, USA). Then, qPCR was performed with Power SYBR Green PCR Master MIX (Applied Biosystems, USA). Glyceraldehyde-3-phosphate dehydrogenase (GAPDH) was used as the internal control. The sequences of primers are listed in Table 1.

**Table 1:** Primers used in qPCR.

Primers	Forward (5’-3’)	Reverse (5’-3’)
GAPDH	AGGTCGGTGTGAACGGATTTG	TGTAGACCATGTAGTTGAGGTCA
CD206	CTCTGTTCAGCTATTGGACGC	CGGAATTTCTGGGATTCAGCTTC
Trem2	CTGGAACCGTCACCATCACTC	CGAAACTCGATGACTCCTCGG
Ym1	CAGGTCTGGCAATTCTTCTGAA	GTCTTGCTCATGTGTGTAAGTGA
Arginase1	CTCCAAGCCAAAGTCCTTAGAG	AGGAGCTGTCATTAGGGACATC
MMP2	ACCTGAACACTTTCTATGGCTG	CTTCCGCATGGTCTCGATG
IL-1β	GCAACTGTTCCTGAACTCAACT	ATCTTTTGGGGTCCGTCAACT
TNF-α	CAGGCGGTGCCTATGTCTC	CGATCACCCCGAAGTTCAGTAG
IL-6	TAGTCCTTCCTACCCCAATTTCC	TTGGTCCTTAGCCACTCCTTC
IL-23	ATGCTGGATTGCAGAGCAGTA	ACGGGGCACATTATTTTTAGTCT

### Statistical analysis

All data were checked for normal distribution and were expressed as mean ± SEM. Differences were analyzed by Student’s *t* test, and a *P* value of less than 0.05 was considered statistically significant. Statistics were performed using GraphPad Prism 7.0 software.

## Results

### *H*. *pylori* infection attenuated the development of CAC

**Fig 2:**
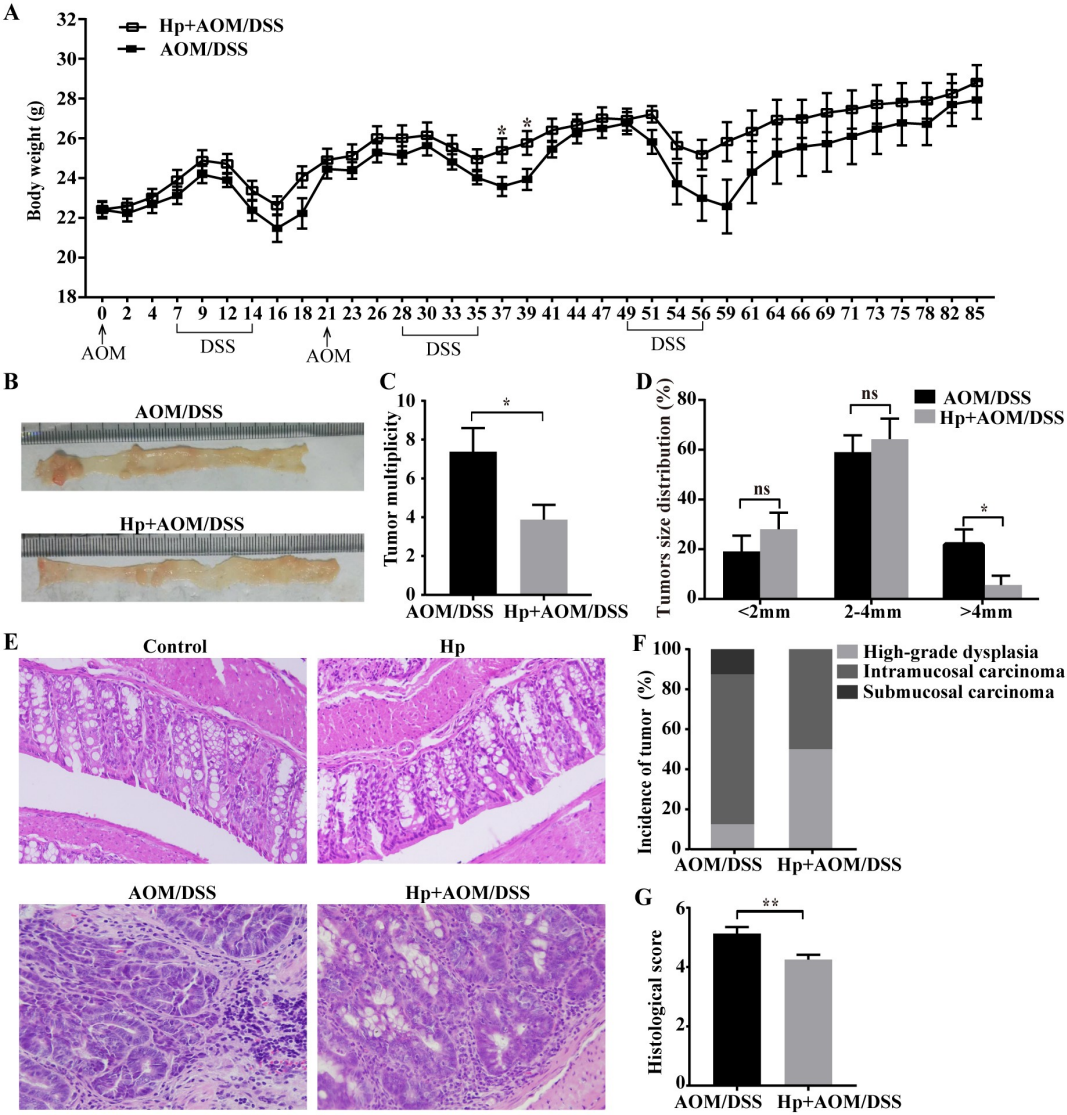
*H*. *pylori* infection attenuated the development of CAC. CAC was induced in mice of AOM/DSS-treated group (AOM/DSS) and AOM/DSS-treated with *H*. *pylori* infection group (Hp+AOM/DSS). (A) Body weight change (n = 8/group). (B) Representative images of colon tumors. (C) Tumor multiplicity (n = 8/group). (D) Tumor size distribution (n = 8/group). (E) Representative images of colonic tissues in control and *H*. *pylori* infection (Hp) group and tumor tissues in AOM/DSS group and Hp+AOM/DSS group by H&E staining (×200). (F) The incidence of each tumor grading (n = 8/group). (G) Histological inflammation scores (n = 8/group). Data are represented as mean ± SEM. *P < 0.05, **P < 0.01, ns, not significant.

Mice’s body weight of each group over time was shown in Fig 2A. During each cycle of DSS administration, mice in AOM/DSS group lost more body weight. All mice developed colitis-associated tumor after 85-day AOM/DSS challenge (Fig 2B). Mice in Hp+AOM/DSS group displayed the significantly decreased tumor multiplicity and low frequency of tumor size (>4mm) compared with AOM/DSS group (Fig 2C and 2D). To further assess the colitis-associated tumor, H&E staining was performed. All tumors were adenomas with high-grade dysplasia or carcinomas (Fig 2E). As shown in Fig 2F, 12.5% (1/8), 75% (6/8) and 12.5% (1/8) of tumors in CAC mice were high-grade dysplasia, intramucosal and submucosal carcinoma respectively, whereas 50% (4/8) and 50% (4/8) of tumors in *H*. *pylori*-infected CAC mice were high-grade dysplasia and intramucosal carcinoma respectively. Moreover, scores of colitis were higher in the AOM/DSS group than in the Hp+AOM/DSS group (Fig 2G), and there were no histological changes in colonic tissues of control group and Hp group. Collectively, these findings suggested that *H*. *pylori* infection attenuated the progression of CAC. In addition, there was only one mouse in Hp+AOM/DSS group with negative anti-*H*. *pylori* staining in gastric tissues, and data from this mouse were discarded.

### *H*. *pylori* infection decreased TAMs infiltration in CAC

**Fig 3:**
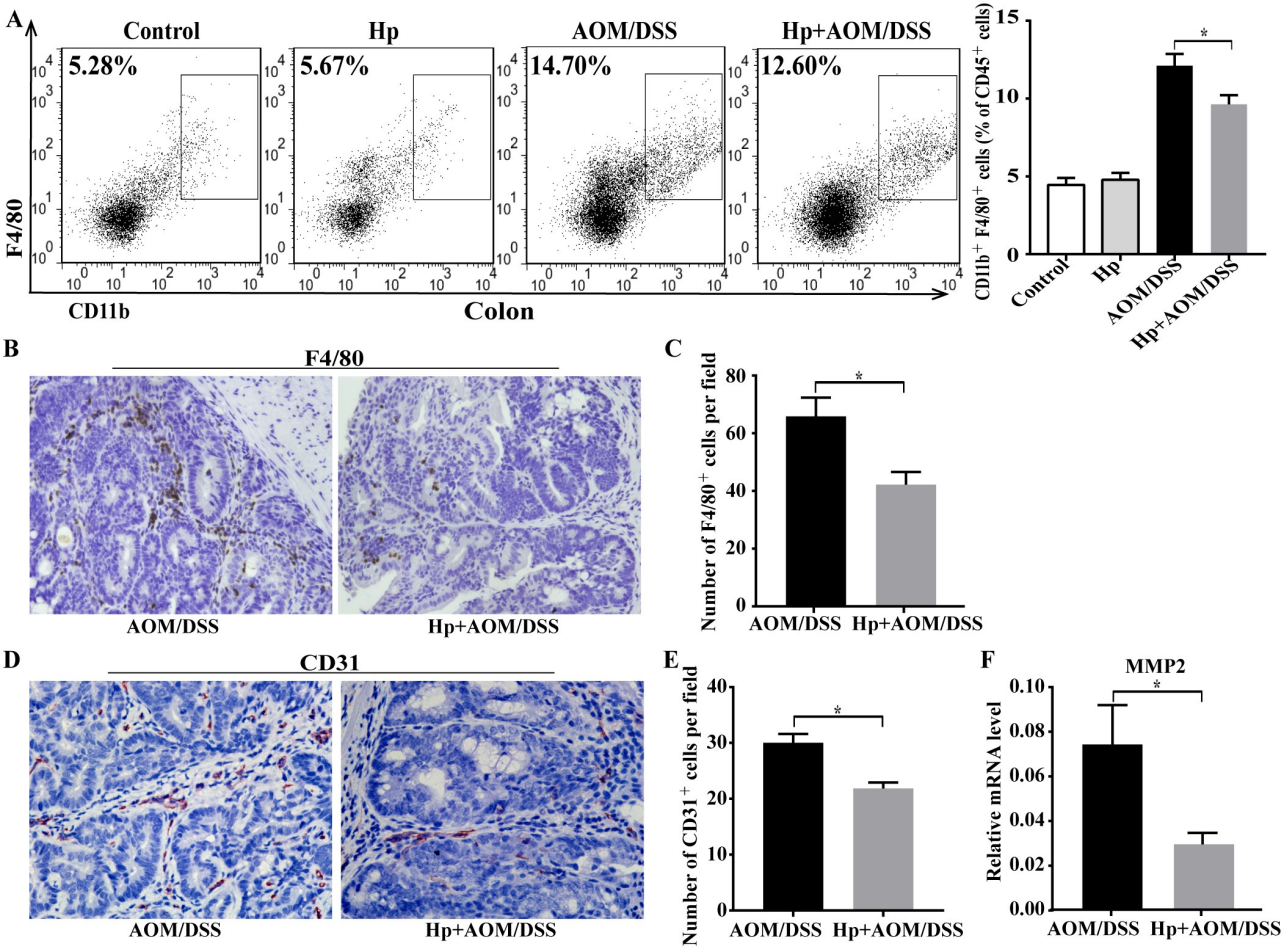
*H*. *pylori* infection decreased TAMs infiltration in CAC. (A) Flow cytometry analysis of CD45^+^CD11b^+^F4/80^+^ TAMs in colonic tissues from AOM/DSS-treated group (AOM/DSS) and AOM/DSS-treated with *H*. *pylori* infection group (Hp+AOM/DSS) (n = 6/group). (B) Representative immunohistochemical images of the TAM marker F4/80 staining in tumor tissues (×400) and (C) the numbers of F4/80^+^ cells per field (n = 6/group). (D) Representative immunohistochemical images of the CD31 staining in tumor tissues (×400) and (E) the numbers of CD31^+^ cells per field (n = 6/group). (F) Relative mRNA expression of MMP2 in colonic tumor tissues (n = 6/group). Data are represented as mean ± SEM. *P < 0.05.

At the end of 85-day CAC challenge, immunocytes were isolated from fresh colonic tissues, and were characterized by flow cytometry. In CAC-bearing mice, the percentages of CD45^+^CD11b^+^F4/80^+^ TAMs were notably increased in colon, but there was a marked reduction of TAMs in Hp+AOM/DSS group when compared with AOM/DSS group (Fig 3A). Then, we further confirmed the results by F4/80 staining. Similar to the results of flow cytometry analysis, immunohistochemical analysis showed that *H*. *pylori*-infected CAC mice significantly decreased the number of F4/80^+^ macrophages in colonic tumors versus CAC mice (Fig 3B and 3C). These results supported that *H*. *pylori* infection diminished the infiltration of TAMs in CAC tumors.

TAMs can promote the tumor invasion and metastasis. Therefore, we assessed the vascular density in by CD31 immunostaining. As shown in Fig 3D and 3E, the expression of CD31 was clearly decreased in tumors of Hp+AOM/DSS group. TAMs could produce matrix metalloproteinases (MMPs) [18]. Thus, our study detected the MMP2 expression in colonic tumors by qPCR. As shown in Fig 3F, *H*. *pylori* infection obviously reduced the mRNA levels of MMP2 in mice after CAC challenge. Thereby *H*. *pylori* infection might limit the progression of CAC.

### *H*. *pylori* infection reduced M2 macrophages proportion in CAC

**Fig 4:**
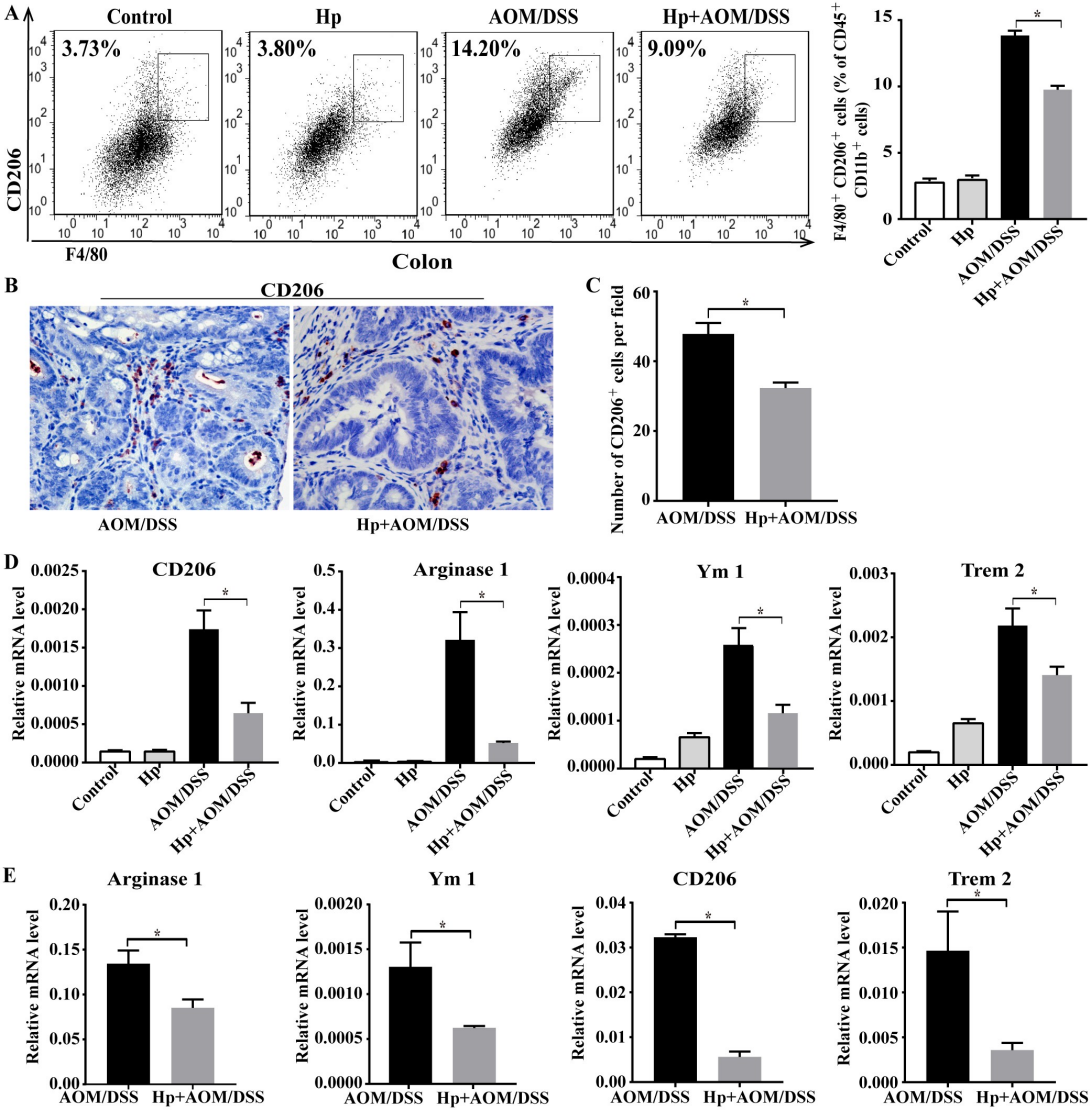
*H*. *pylori* infection reduced M2 macrophages proportion in CAC. (A) Flow cytometry analysis of CD45^+^CD11b^+^F4/80^+^CD206^+^ M2 macrophages in colonic tissues from AOM/DSS-treated group (AOM/DSS) and AOM/DSS-treated with *H*. *pylori* infection group (Hp+AOM/DSS) (n = 6/group). (B) Representative immunohistochemical images of the M2 marker CD206 staining in tumor tissues (×400) and (C) the numbers of CD206^+^ cells per field (n = 6/group). (D) Relative mRNA expression of M2-associated genes (Arginase1, CD206, Trem2 and Ym1) in colonic tumors (n = 5-6/group). (E) Arginase1, Ym1, CD206 and Trem2 mRNA expression in F4/80^+^ macrophages isolated from tumors of AOM/DSS group and Hp+AOM/DSS group (n = 5-6/group). Data are represented as mean ± SEM. *P < 0.05.

As M2 macrophages play a pro-tumor role in colon cancer, we explored the effect of *H*. *pylori* infection on them and used a common marker CD206 to identify M2 subtype from total macrophages. As shown in Fig 4A, *H*. *pylori*-infected CAC mice exhibited lower percentages of CD45^+^CD11b^+^F4/80^+^CD206^+^ M2 macrophages in colon than CAC mice. Consist with the decreased proportion of M2 macrophages, the CD206 expression in tumor by immunostaining and the mRNA levels of representative M2 genes (Arginase1, CD206, Trem2 and Ym1) by qPCR were also markedly down-regulated in colonic tumors of *H*. *pylori*-infected CAC mice (Fig 4B–4D).

To further confirm the effect of *H*. *pylori* infection on the M2 phenotype of TAMs in CAC, we isolated F4/80^+^ TAMs from colonic tumor tissues and analyzed the M2-associated genes expression. As shown in Fig 4E, the mRNA levels of Arginase1, Ym1, CD206 and Trem2 were significantly decreased in TAMs from *H*. *pylori*-infected CAC mice compared with TAMs from CAC mice. Taken together, these findings indicated that *H*. *pylori* infection reduced the infiltration of M2-like TAMs in CAC tumor.

### *H*. *pylori* infection regulated the pro-inflammatory and pro-tumorigenic cytokines expression in CAC

**Fig 5:**
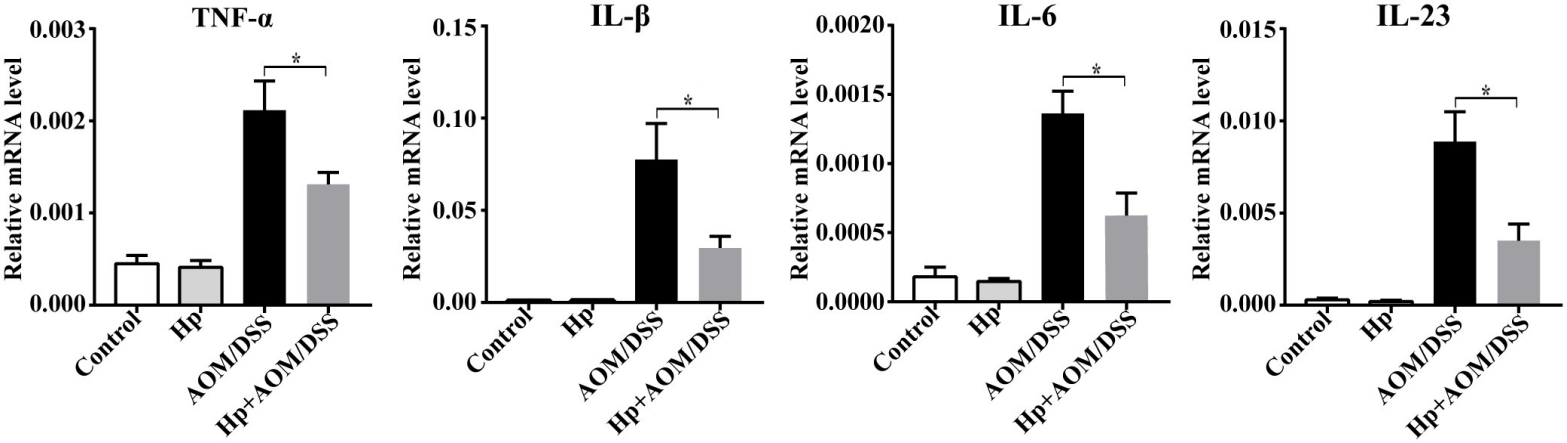
*H*. *pylori* infection regulated the pro-inflammatory and pro-tumorigenic cytokines expression in CAC mice. Relative mRNA expression of pro-inflammatory and pro-tumorigenic cytokines (TNF-α, IL-1β, IL-6 and IL-23) in colonic tumors (n = 5-6/group). Data are represented as mean ± SEM. *P < 0.05.

To further explore the effect of *H*. *pylori* infection on the function of TAMs in CAC, we investigated mRNA expression of pro-inflammatory and pro-tumorigenic cytokines, which are associated with TAMs. In accordance with the decreased TAMs proportion, mRNA levels of TNF-α, IL-1β, IL-6 and IL-23 were also obviously down-regulated in tumors of *H*. *pylori*-infected CAC mice. Thus, *H*. *pylori* infection appears to affect the TAMs in CAC through regulating the cytokines expression (Fig 5).

## Discussion

In this study, we have revealed that *H*. *pylori* infection attenuated the development of CAC by decreasing tumor multiplicity and tumor size. Histologically, *H*. *pylori* infection also slowed down the colitis-associated tumor progression, supported by tumor grading and colitis scores. Moreover, *H*. *pylori* infection reduced infiltration of TAMs, particularly M2-like TAMs in CAC tumors. This effect might be associated with the down-regulated pro-inflammatory and pro-tumorigenic cytokines in colonic tumors of *H*. *pylori*-infected CAC mice.

Accumulations of TAMs is common in tumors, including colon cancer [19, 20]. TAMs also include two subtypes: M1 and M2. At the early stage of tumor development, TAMs exhibit M1-like phenotype, which promote immune responses that kill tumor cells. During the late stage of tumor progression, TAMs generally switch to M2-like phenotype, which exert tumor-promoting activities [13, 21]. In inflammatory and tumor microenvironment, TAMs can produce cytokines such as IL-6/IL-17/IL-23 to induce the tumor initiation and progression via the NF-κB or STAT3 signaling pathway. TAMs-derived TNF-α can transport to destination organs, which promote tumor cells recruitment in metastatic foci [22]. Evidences also suggest that TAMs promote tumor metastasis through producing matrix metallopeptidase (MMP), such as MMP2, MMP7, and MMP9 [18]. Previous study has showed that VEGF, TNF-α, IL-1β and MMPs derived from TAMs involve in tumor angiogenesis [23].

In an AOM/DSS-induced CAC mouse model, the percentages of CD68^+^ macrophages and CD206^+^ M2 macrophages increased in tumors, accompanied with the up-regulated expression of TNF-α, IL-1β, IL-6 [24]. Studies revealed that anti-tumor drugs inhibited the M2-associated genes, such as CD206, Arginase 1, CD204 and MMP2 in AOM/DSS mouse models [25, 26]. The inflammatory cytokines, including TNF-α, IL-1β and IL-6, were also suppressed and the percentages of macrophages were decreased, after using a kind of anti-ulcerative colitis medicine in CAC [27].

Evidences have demonstrated that *H*. *pylori* infection decreased the pro-inflammatory cytokines (TNF-α, IFN-γ, IL-1β, IL- 6, IL-17A and IL23) in colitis mouse models [6, 10], and we also have reported that IL-6 and IL23 expression were down-regulated in *H*. *pylori*-infected chronic colitis mice. In present study, we have found that *H*. *pylori* infection significantly reduced the proportion of TAMs and the expression of pro-inflammatory and pro-tumorigenic cytokines (TNF-α, IL-1β, IL-6 and IL-23) in a CAC model, suggesting that *H*. *pylori* infection might affect TAMs by regulating the cytokine milieu in CAC mice. *H*. *pylori* infection also decreased the MMP2 expression related to TAMs, and thereby *H*. *pylori* infection can limit colonic tumor metastasis. Moreover, we also found the infiltration of M2 TAMs were diminished in *H*. *pylori* infected CAC, suggesting that *H*. *pylori* infection appears to attenuate CAC through regulating differentiation of TAMs. However, the specific mechanism about how *H*. *pylori* infection regulates the TAMs in CAC remains unknown.

In Conclusion, our study suggests that *H*. *pylori* infection reduces TAMs infiltration especially M2-like TAMs and regulates the cytokines expression in CAC.

## References

[1] Lin KD, Chiu GF, Waljee AK, Owyang SY, El-Zaatari M, Bishu S, et al Effects of Anti-Helicobacter pylori Therapy on Incidence of Autoimmune Diseases, Including Inflammatory Bowel Diseases. Clin Gastroenterol Hepatol. 2019;17(10):1991–9. 10.1016/j.cgh.2018.12.014 . 30580094 PMC9629375

[2] Eaden JA, Abrams KR, Mayberry JF. The risk of colorectal cancer in ulcerative colitis: a meta-analysis. Gut. 2001;48(4):526–35. 10.1136/gut.48.4.526 . 11247898 PMC1728259

[3] Canavan C, Abrams KR, Mayberry J. Meta-analysis: colorectal and small bowel cancer risk in patients with Crohn’s disease. Aliment Pharmacol Ther. 2006;23(8):1097–104. 10.1111/j.1365-2036.2006.02854.x . 16611269

[4] Ullman TA, Itzkowitz SH. Intestinal inflammation and cancer. Gastroenterology. 2011;140(6):1807–16. 10.1053/j.gastro.2011.01.057 . 21530747

[5] Li X, Tan J, Zhang F, Xue Q, Wang N, Cong X, et al H. pylori Infection Alleviates Acute and Chronic Colitis with the Expansion of Regulatory B Cells in Mice. Inflammation. 2019;42(5):1611–21. 10.1007/s10753-019-01022-0 . 31377948

[6] Wu YZ, Tan G, Wu F, Zhi FC. H. pylori attenuates TNBS-induced colitis via increasing mucosal Th2 cells in mice. Oncotarget. 2017;8(43):73810–6. 10.18632/oncotarget.17962 . 29088747 PMC5650302

[7] Choi DS, Seo SI, Shin WG, Park CH. Risk for Colorectal Neoplasia in Patients With Helicobacter pylori Infection: A Systematic Review and Meta-analysis. Clin Transl Gastroenterol. 2020;11(2):e00127 10.14309/ctg.0000000000000127 . 32032128 PMC7145030

[8] Zhang Y, Hoffmeister M, Weck MN, Chang-Claude J, Brenner H. Helicobacter pylori infection and colorectal cancer risk: evidence from a large population-based case-control study in Germany. Am J Epidemiol. 2012;175(5):441–50. 10.1093/aje/kwr331 . 22294430

[9] Luo C, Zhang H. The Role of Proinflammatory Pathways in the Pathogenesis of Colitis-Associated Colorectal Cancer. Mediators Inflamm. 2017;2017:5126048 10.1155/2017/5126048 . 28852270 PMC5568615

[10] Zhang H, Dai Y, Liu Y, Wu T, Li J, Wang X, et al Helicobacter pylori Colonization Protects Against Chronic Experimental Colitis by Regulating Th17/Treg Balance. Inflamm Bowel Dis. 2018;24(7):1481–92. 10.1093/ibd/izy107 . 29788098

[11] Ostuni R, Kratochvill F, Murray PJ, Natoli G. Macrophages and cancer: from mechanisms to therapeutic implications. Trends Immunol. 2015;36(4):229–39. 10.1016/j.it.2015.02.004 . 25770924

[12] Dehne N, Mora J, Namgaladze D, Weigert A, Brune B. Cancer cell and macrophage cross-talk in the tumor microenvironment. Curr Opin Pharmacol. 2017;35:12–9. 10.1016/j.coph.2017.04.007 . 28538141

[13] Chanmee T, Ontong P, Konno K, Itano N. Tumor-associated macrophages as major players in the tumor microenvironment. Cancers (Basel). 2014;6(3):1670–90. 10.3390/cancers6031670 . 25125485 PMC4190561

[14] Yunna C, Mengru H, Lei W, Weidong C. Macrophage M1/M2 polarization. Eur J Pharmacol. 2020;877:173090 10.1016/j.ejphar.2020.173090 . 32234529

[15] Dai Y, Jiao H, Teng G, Wang W, Zhang R, Wang Y, et al Embelin reduces colitis-associated tumorigenesis through limiting IL-6/STAT3 signaling. Molecular cancer therapeutics. 2014;13(5):1206–16. 10.1158/1535-7163.MCT-13-0378 . 24651526

[16] Zaki MH, Vogel P, Malireddi RK, Body-Malapel M, Anand PK, Bertin J, et al The NOD-like receptor NLRP12 attenuates colon inflammation and tumorigenesis. Cancer Cell. 2011;20(5):649–60. 10.1016/j.ccr.2011.10.022 . 22094258 PMC3761879

[17] Weigmann B, Tubbe I, Seidel D, Nicolaev A, Becker C, Neurath MF. Isolation and subsequent analysis of murine lamina propria mononuclear cells from colonic tissue. Nat Protoc. 2007;2(10):2307–11. 10.1038/nprot.2007.315 . 17947970

[18] Deryugina EI, Quigley JP. Tumor angiogenesis: MMP-mediated induction of intravasation- and metastasis-sustaining neovasculature. Matrix Biol. 2015;44–46:94–112. 10.1016/j.matbio.2015.04.004 . 25912949 PMC5079283

[19] Mantovani A, Marchesi F, Malesci A, Laghi L, Allavena P. Tumour-associated macrophages as treatment targets in oncology. Nat Rev Clin Oncol. 2017;14(7):399–416. 10.1038/nrclinonc.2016.217 . 28117416 PMC5480600

[20] Szebeni GJ, Vizler C, Kitajka K, Puskas LG. Inflammation and Cancer: Extra- and Intracellular Determinants of Tumor-Associated Macrophages as Tumor Promoters. Mediators Inflamm. 2017;2017:9294018 10.1155/2017/9294018 . 28197019 PMC5286482

[21] Ngambenjawong C, Gustafson HH, Pun SH. Progress in tumor-associated macrophage (TAM)-targeted therapeutics. Adv Drug Deliv Rev. 2017;114:206–21. 10.1016/j.addr.2017.04.010 . 28449873 PMC5581987

[22] Yang L, Zhang Y. Tumor-associated macrophages: from basic research to clinical application. J Hematol Oncol. 2017;10(1):58 10.1186/s13045-017-0430-2 . 28241846 PMC5329931

[23] Qian BZ, Pollard JW. Macrophage diversity enhances tumor progression and metastasis. Cell. 2010;141(1):39–51. 10.1016/j.cell.2010.03.014 . 20371344 PMC4994190

[24] Wang W, Li X, Zheng D, Zhang D, Peng X, Zhang X, et al Dynamic changes and functions of macrophages and M1/M2 subpopulations during ulcerative colitis-associated carcinogenesis in an AOM/DSS mouse model. Mol Med Rep. 2015;11(4):2397–406. 10.3892/mmr.2014.3018 . 25434400 PMC4337491

[25] Li H, Li L, Mei H, Pan G, Wang X, Huang X, et al Antitumor properties of triptolide: phenotype regulation of macrophage differentiation. Cancer Biol Ther. 2020;21(2):178–88. 10.1080/15384047.2019.1679555 . 31663424 PMC7012063

[26] Wu T, Dai Y, Wang W, Teng G, Jiao H, Shuai X, et al Macrophage targeting contributes to the inhibitory effects of embelin on colitis-associated cancer. Oncotarget. 2016;7(15):19548–58. 10.18632/oncotarget.6969 . 26799669 PMC4991400

[27] Lin X, Yi Z, Diao J, Shao M, Zhao L, Cai H, et al ShaoYao decoction ameliorates colitis-associated colorectal cancer by downregulating proinflammatory cytokines and promoting epithelial-mesenchymal transition. J Transl Med. 2014;12:105 10.1186/1479-5876-12-105 . 24766737 PMC4022058

